# CINET: A Brain-Inspired Deep Learning Context-Integrating Neural Network Model for Resolving Ambiguous Stimuli

**DOI:** 10.3390/brainsci10020064

**Published:** 2020-01-24

**Authors:** Rajesh Amerineni, Resh S. Gupta, Lalit Gupta

**Affiliations:** 1 Department of Electrical & Computer Engineering, Southern Illinois University, Carbondale, IL 62901, USA; rajeshamerineni@siu.edu; 2 Vanderbilt Brain Institute, Vanderbilt University, Nashville, TN 37232, USA; resh.s.gupta@vanderbilt.edu

**Keywords:** Context effect, deep learning, convolution neural networks, ambiguous stimuli

## Abstract

The brain uses contextual information to uniquely resolve the interpretation of ambiguous stimuli. This paper introduces a deep learning neural network classification model that emulates this ability by integrating weighted bidirectional context into the classification process. The model, referred to as the CINET, is implemented using a convolution neural network (CNN), which is shown to be ideal for combining target and context stimuli and for extracting coupled target-context features. The CINET parameters can be manipulated to simulate congruent and incongruent context environments and to manipulate target-context stimuli relationships. The formulation of the CINET is quite general; consequently, it is not restricted to stimuli in any particular sensory modality nor to the dimensionality of the stimuli. A broad range of experiments is designed to demonstrate the effectiveness of the CINET in resolving ambiguous visual stimuli and in improving the classification of non-ambiguous visual stimuli in various contextual environments. The fact that the performance improves through the inclusion of context can be exploited to design robust brain-inspired machine learning algorithms. It is interesting to note that the CINET is a classification model that is inspired by a combination of brain’s ability to integrate contextual information and the CNN, which is inspired by the hierarchical processing of information in the visual cortex.

## 1. Introduction

**Figure 1:**
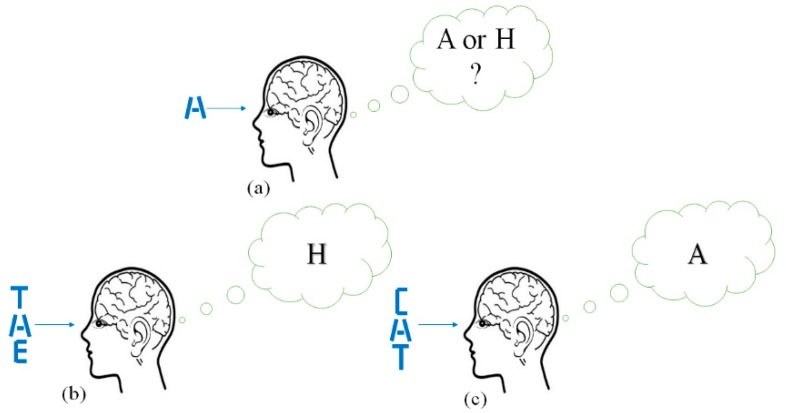
Interpreting an ambiguous letter (**a**) without context, (**b**) with context T and E, and (**c**) with context C and T.

The goal of this paper is to develop a versatile deep learning neural network classification model that improves the interpretation of ambiguous and degraded stimuli through the inclusion of context during the training and testing phases. The deep learning neural network selected for the classification model is the convolution neural network (CNN) because it offers an effective way to integrate context stimuli with a target stimulus for the purpose of extracting features that are coupled across the target and context stimuli. The resulting context-integrating CNN classification model is referred to as the CINET. The CINET is inspired by the context effect, which is the influence of the surrounding environment on the perception of stimuli [1,2,3]. Numerous studies related to the context effect have shown that the integration of contextual information improves the interpretation of spoken words [4,5], written letters and words [6,7,8], physical objects [9,10,11], sounds [12,13], smells [14], tastes [15], threats [16], colors [17], and facial emotions [18,19,20]. The context effect has also been widely studied to show how contextual information is used to uniquely resolve the interpretation of ambiguous stimuli [7,8,21,22,23,24,25,26]. Ambiguous stimuli contain conflicting sensory information which provides the brain with multiple, mutually exclusive interpretations [24]. Figure 1 is a simplified illustration of an example that is often used to show how the brain exploits contextual information to correctly interpret an ambiguous letter. In isolation, the letter in Figure 1a is equally likely to be interpreted as an A or an H. However, as shown in Figure 1b,c the same ambiguous letter is uniquely interpreted as an H in the word THE and as an A in the word CAT.

The CINET attempts to emulate the brain’s ability to resolve the interpretation of ambiguous and degraded stimuli; however, it is not aimed at modelling the internal mechanisms of the brain involved in context integration. Instead, the aim is to model, at the input-output level, how context included in the learning phase influences the resolution of stimuli in the classification phase. Specifically, the goal is to demonstrate that the CINET parameters can be manipulated to emulate various aspects of the Context Shift Decrement (CSD) principle [27] and the related Context Reinstatement Effect (CRE) [28], which are central to explaining how context influences perception. Together, the CSD and CRE principle state that recognition is more accurate if the relationship between the context and target is strong, and recognition decreases when this relationship is weak or the context is changed during the recognition phase. A letter classification problem is selected because it can elegantly demonstrate the capabilities and performance of the CINET by incorporating context letters to form meaningful words. The model, however, is equally applicable to more complex problems, such as the interpretation of ambiguous objects in the visual domain and ambiguous words in a spoken sentences in the auditory domain. Furthermore, the target and context stimuli can be from different modalities to emulate multisensory context integration.

The structure of the paper is as follows: Section 2 describes the structure and parameters of the generalized CINET classifier model. The CNN implementation of the CINET for multidimensional inputs is described in Section 3. The visual stimuli used in the experiments and the methods used to manipulate target and context stimuli are described in Section 4. The series of experiments designed to demonstrate the capabilities and properties of the CINET, the results, and a discussion of the results are presented in Section 5. Finally, the contributions of the study are summarized in Section 6.

## 2. The Generalized CINET Classifier Model

The interpretation of ambiguous stimuli is formulated as a pattern recognition problem; therefore, the focus is on modelling the mapping between an ambiguous stimulus (system input) and the class of the ambiguous stimulus (system output). Due to the inclusion of context, the design of the CINET classifier is unlike the design of most pattern classifiers, which mainly focus on training and testing with isolated, context-free patterns. In the formulations, the stimuli classes are represented by *ω_i_,i* = 1, 2, …, *L*, where L is the number of stimulus classes.

**Figure 2:**
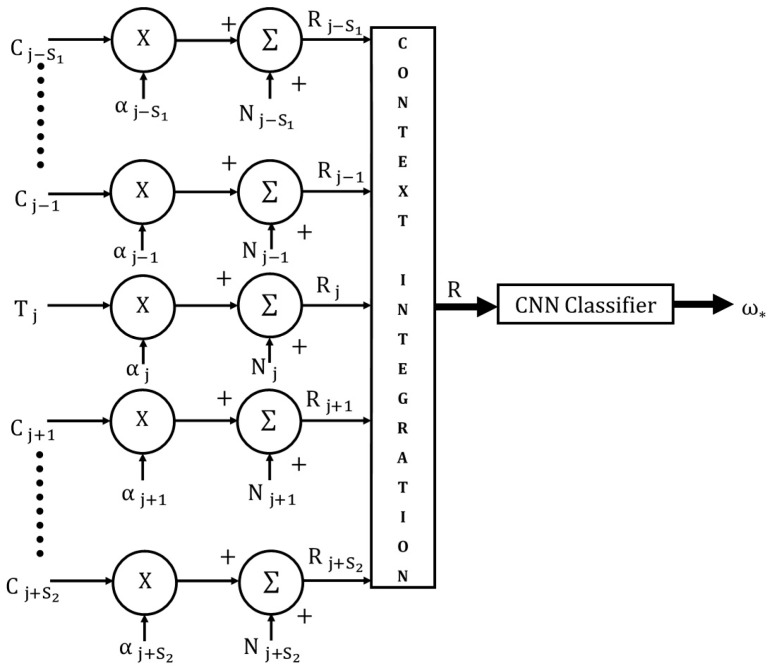
The generalized CINET(S) classifier model.

The proposed CINET classifier model is illustrated in Figure 2. This section focuses on the input and context integration component of the model. The CNN classifier component is described in detail in the next section. In the model, the target stimulus is represented by T, the context stimuli by C, the context weights by α, the stimulus noise by N, the weighted and noisy stimuli by $R_{j}$, the context-integrated stimulus by R, and the classifier output by $\omega_{*}$. The context-integrated stimulus, which is the input to the CNN classifier, can be written as

(1) $$R=[\left(\alpha_{j-S_{1}}C_{j-S_{1}}+N_{j-S_{1}}\right)\nabla \ldots \nabla \left(\alpha_{j-2}C_{j-2}+N_{j-2}\right)\nabla \left(\alpha_{j-1}C_{j-1}+N_{j-1}\right)\nabla \left(\alpha_{j}T_{j}+N_{j}\right),\;\left(\alpha_{j+1}C_{j+1}+N_{j+1}\right)\nabla \left(\alpha_{j+2}C_{j+2}+N_{j+2}\right)\nabla \ldots \nabla \left(\alpha_{j+S_{2}}C_{j+S_{2}}+N_{j+S_{2}}\right)],$$

where the symbol ∇ is used to represent the general context-integration operation. Equation (1) can be written more compactly as

(2) $$R=\nabla_{i=-S_{1}}^{S_{2}}R_{j+i},$$

where, $R_{j+i}=\left(\alpha_{j}T_{j}+N_{j}\right)$ when i=0 and $R_{j+i}=\left(\alpha_{j+i}C_{j+i}+N_{j+i}\right)$ when i≠0. 

In this generalized formulation, the subscript j can represent a position (spatial) index or a time (temporal) index. The transformed target stimulus is padded on the left and right by $S_{1}$ and $S_{2}$ transformed context stimuli where both $S_{1}$ and $S_{2}$ are positive constants with values greater than or equal to zero. The context “span” is defined as S, where, $S=S_{1}+S_{2}$, and the resulting classifier is referred to as a CINET(S) classifier. The model is symmetrical if $S_{1}=S_{2}$ and asymmetrical if $S_{1}\neq S_{2}$. Furthermore, if $S_{1}=S_{2}=0$, that is, the context span S=0, the CINET(S) classifier reduces to a context-free classifier represented by CINET(0). For the CINET(0) classifier, the input stimulus is simply $\left(\alpha_{j}T_{j}+N_{j}\right)$. The weight $\alpha_{j+i}$ assigned to context $C_{j+i}$ can be varied from zero (no influence) to one (full influence) in order to control the strength of the target-context relationship. The noise $N_{j}$ and $N_{j+i}$ added to $T_{j}$ and $C_{j+i}$ accounts for randomness in the target and context stimuli, respectively.

The context-integration operation ∇ in Equation (2) is critical because it specifies the manner in which the target and context stimuli are integrated to form the input into the CNN classifier, which in turn will determine the type of features that are extracted from the context-integrated input. For example, if the target T and the context stimuli C are H×W arrays, they can be integrated into an H×W array through averaging, a large (M)(H)×W array through concatenation, or an H×W×(S+1) cuboid through a stacking operation. The averaging operation mixes the target and context stimulus arrays into a single array. As a result, there is no control over the strength of the coupling between the target and context stimuli. The concatenation operation also suffers from a lack of controlled coupling. The cuboid option is selected for the development of the CINET classifier model because it offers the most flexible choices for selecting features that are not only coupled across the target and context stimuli, but also features with controlled coupling.

## 3. CNN Implementation of the CINET Classifier Model

In the most general case, the CNN classifier in Figure 2 can be replaced with any classifier. As noted in the Introduction, the CNN is selected because it is ideal for combining target and context stimuli and for extracting coupled target-context features with controlled coupling. This section begins with a brief introduction to CNNs and is followed by a detailed description of the multidimensional CINET and its special cases.

### 3.1. Convolution Neural Networks

CNNs, inspired by the pioneering work of Nobel laureates David Hubel and Thorsten Wiesel on information processing in the visual cortex [29,30,31], are a class of deep learning networks that have proven to be very effective for large-scale object classification and detection in images [32,33,34,35,36,37,38]. Common CNN architectures generally consist of a series of convolution and pooling layers followed by a fully connected network (FCN). The function of the convolution operations in each layer is to detect features from the output of the previous layer. As a result, the complexity of the features detected increases as the number of convolution layers in the network increases. The pooling layer reduces the spatial dimension of the convolution layer output through subsampling. The most often used pooling operation is max-pooling, in which a block of features is replaced by its maximum value in order to select the most robust feature in the block. The FCN is a standard feed-forward network using either sigmoid or tanh activation functions in the hidden layers and softmax activations in the outputs in order to interpret the network outputs as class posterior probabilities. The gradient descent backpropagation algorithm is used to train the network.

Designing a CNN for a given problem involves specifying the architecture, which includes the number of convolution layers; the number, stride, padding, and the dimensions of the filters in each convolution layer, the size, stride, operation (maximum, average) of the filters in the pooling layers; the sequence of the convolution and pooling layers; the number of layers in the FCN; and the activation functions in the convolution and FCN layers. The hyperparameters that need to be specified during the training phase include the loss function, weight-initialization, learning rate, momentum term, convergence criterion, and batch size.

### 3.2. The Multidimensional CINET(S) Model

The most general formulation of the CINET(S) in Figure 2 is obtained by assuming that stimuli T and C are multidimensional (arrays with more than two dimensions). A color image comprised of red, green, and blue component images may be regarded as a three-dimensional stimulus. Examples of three-dimensional signals include seismic volumes, X-ray computed tomography, and LIDAR data. In the generalized formulation, the multidimensional input into the CNN can include higher-dimensional arrays, such as multisensor satellite images and hyperspectral images. Each multidimensional input can be represented by a cuboid, and the cuboids from multiple inputs can be integrated, using the stacking operation, into hypercuboids. The height, width, and depth of hypercuboids and cuboids will be represented by the variables h, w, and z, respectively. Note that z does not represent the depth (number of layers) of the CNN. To avoid this confusion, the cuboid depth will be referred to as “*z*-depth.”

**Figure 3:**
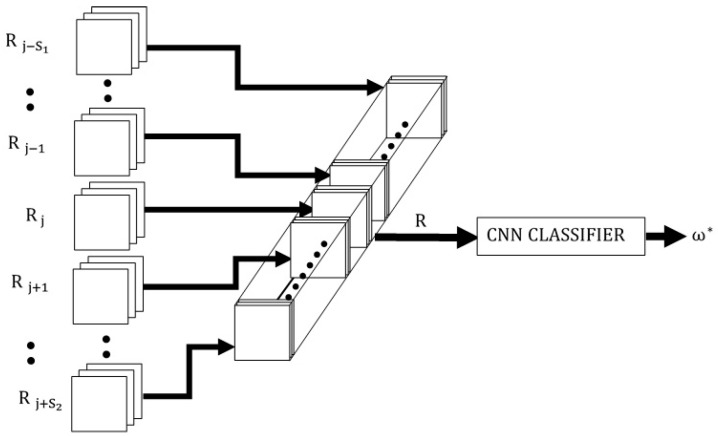
The multidimensional CINET(S) classifier model.

The CINET(S) for multidimensional stimuli, shown in Figure 3, is described in detail. It is then shown that the models for one-dimensional and two-dimensional stimuli are special cases of the multidimensional stimulus model. If H, W, and $Z_{j}$ are the height, width, and z-depth of the multidimensional input stimuli, respectively, the dimension of the cuboid $R_{j}$ in Equation (2) will be $H\times W\times Z_{j}$, and the dimension of the hypercuboid R in Equation (1) will be $H\times W\times Z=Z_{j}\left(1+S\right).$ That is, the input to the CNN is the hypercuboid R(h,w,z) of dimension H×W×Z formed by stacking the weighted-noisy target and context stimuli, as shown in the figure.

In order to simplify the formulations, it is assumed that the convolutions in each layer are the “same” through zero-padding the input so that the filter outputs have the same dimensions as the input. Moreover, it will be assumed that the height and width of the filters in all convolution layers are the same. If the convolution is “valid,” the dimensions of the filtered outputs can be easily adjusted according to the height and width of the filter. In the first convolution layer, each filter is selected to be a cuboid filter with the same z-depth as the input hypercuboid so that the target and context are fully coupled within the receptive field of each neuron in the layer. The filters, centered at zero in the (h,w) plane, are assumed to have dimensions [(2a+1)×(2b+1)×Z]. If the number of filters in the first layer is $K_{1}$ and the kth cuboid filter is represented by $f^{\left[1,k\right]}\left(x,y,z\right),x=-a,\ldots ,0,\ldots ,a;\;y=-b,\ldots 0,\ldots ,b;z=0,1,\ldots ,\left(Z-1\right);k=0,1,\ldots ,\left(K_{1}-1\right)$, the output of the filter is given by

$${\hat{R}}^{\left[1,k\right]}\left(h,w\right)=\sum_{z=0}^{Z-1}\sum_{u=-a}^{a}\sum_{v=-b}^{b}f^{\left[1,k\right]}\left(u,v,z\right)R\left(h+u,w+v,z\right),\;\;h=0,1,\ldots ,(H-\;1);\;w=0,1,\ldots ,\left(W-1\right).$$

Note that the convolution of the input hypercuboid with a cuboid filter having the same z-depth results in an array with dimension H×W. A bias $B^{\left[1,k\right]}$ is added to the filtered output and passed through the nonlinear ReLu activation function so that the activation of filter k in the first layer is given by

$${\tilde{R}}^{\left[1,k\right]}\left(h,w\right)=\;ReLu[{\hat{R}}^{\left[1,k\right]}\left(h,w\right)+\;B^{\left[1,k\right]}],$$

where, ReLu[δ]=Max[0,δ]. The output of the first convolution layer are the $K_{1}$ activations combined into a $H\times W\times K_{1}$ cuboid, which can be written as

$R^{\left[1\right]}\left(h,w,k\right)=\nabla_{k=0}^{K_{1}-1}{\tilde{R}}^{\left[1,k\right]}\left(h,w\right)$, $h=0,1,\ldots ,\left(H-1\right),\;w=0,1,\ldots ,\left(W-1\right),\;k=0,1,\ldots ,\left(K_{1}-1\right).$ If pooling follows and the stride and size of the pooling filter are ∝ and (γ× γ×1), respectively, the output of the pooling layer is given by

R[1,p](h,w,k)= [R[1](hp,wp,k)](hp,wp)∈GhpMAX, h=0,1,…,(H[1]−1),w=0,1,…,(W[1]−1),k=0,1,…,(K1−1)

where,$\;G_{hp}=\{\left(\propto \;\times h+t_{h},\propto \;\times w+t_{w}\right),\;t_{h},\;t_{w}=0,1,\ldots ,\left(\gamma -1\right)$} is the pooling window, and $H^{\left[1\right]}=\left(H-\gamma \right)/\propto$+1, and $W^{\left[1\right]}=\left(W-\gamma \right)/\propto$+1 are the height and width of the pooled output, respectively. In the next convolution stage, the cuboid $R^{\left[1,p\right]}\left(h,w,k\right)$ is convolved with a cuboid filter $f^{\left[2,k\right]}\left(x,y,z\right),x=-a,\ldots ,0,\ldots ,a;\;y=-b,\ldots 0,\ldots ,b;z=0,1,\ldots ,\left(K_{1}-1\right);k=0,1,\ldots ,\left(K_{2}-1\right)$ and the filtered output is given by the cuboid convolution

$$R^{\left[2,k\right]}\left(h,w\right)=\overset{K_{1}-1}{\underset{z=0}{\sum }}\overset{a}{\underset{u=-a}{\sum }}\overset{b}{\underset{v=-b}{\sum }}f^{\left[2,k\right]}\left(u,v,z\right)R^{\left[1,p\right]}\left(h+u,w+v,z\right),\;h=0,1,\ldots ,(H^{\left[1\right]}-\;1);\;\;w=0,1,\ldots ,\left(W^{\left[1\right]}-1\right).$$

As in the previous step, a bias is added to each filtered output and passed through the ReLu activation function, and the $K_{2}$ activations are combined into a $H^{\left[1\right]}\times W^{\left[1\right]}\times K_{2}$ cuboid. If a pooling layer follows, the height and width of the cuboid are adjusted accordingly. The convolution and pooling operations are repeated and terminate into a flattening operation in which the rows of the cuboid are combined into a vector which is the input to a fully connected feed-forward neural network with N layers.

The fully connected network (FCN) uses the ReLu, *sigmoidal*, or *tanh* activation function for the intermediate hidden layers, the softmax activation function for the output layer, and the cross-entropy for the loss-function. As noted earlier, it is assumed that the target stimuli $T_{j}$ belongs to one of L classes represented by $\omega_{i},\;i=1,2,\ldots ,L$. The softmax layer will, therefore, have L outputs, one for each class of the target stimulus. If $q_{i}$ is the weighted sum of the inputs into a neuron i in the softmax layer, the softmax layer outputs are given by

$$p\left(\omega_{i}\right)=\frac{e^{q_{i}}}{\sum_{i=1}^{L}e^{q_{i}}}\;,\;i=1,2,\ldots ,L,$$

The cross-entropy cost function is given by

$$E=-\overset{L}{\underset{i=1}{\sum }}t_{i}\log(p\left(\omega_{i}\right)),\;\mathrm{where},\;t_{i}=\{\begin{array}{c} 1\;if\;T_{j}\;\epsilon \omega_{i}\\ 0\;otherwise \end{array}$$

During testing, the softmax outputs can be regarded as estimates of class posterior probabilities; therefore, the target stimulus can be assigned to the class $\omega_{*}$ yielding the highest posterior probability, which is given by

(3) $$\omega_{*}=\arg\;\max\left[\;p\left(\omega_{i}\right)\right],\;i=1,2,\ldots L.$$

### 3.3. Special Cases of the CINET(S) Model

As mentioned earlier, the one-dimensional and two-dimensional inputs into the CNN are special cases of the multidimensional inputs. For the two-dimensional case, the main difference is that z-depth of the target and context stimuli is unity. Therefore, the dimension of $R_{j}$ in Equation (2) will be H×W, and the dimension of the cuboid R in Equation (1) will be H×W×(1+S). The cuboid input to the CNN can, therefore, be written as R(h,w,z), h=0,1,…,(H−1);w=0,1,…,(W−1);z=0,1…,S. In order to match the z-depth of the input cuboid, the filters in the first convolution layer will, therefore, have dimension [(2a+1)×(2b+1)×(1+S)]. Other than the changes in the dimensions of the cuboid input and filters in the first layer, the convolution, pooling, and FCN layer operations are identical to the operations in the multidimensional input case.

For one-dimensional inputs, the heights and depths of the target and context stimuli are unity and are, therefore, vectors. The dimension of $R_{j}$ in Equation (2) will be W, and the dimension of R in Equation (1) will be 1×W×(1+S). Note that, although R is an array, it is written as a cuboid with unity height for consistency. The cuboid input to the CNN can, therefore, be written as R(0,w,z), w=0,1,…,(W−1);z=0,1…,S. The dimension of each filter in the first layer will be 1×(2b+1)×(1+S) and the filtered output will be a vector with dimension W, which can be written as a 1×W×1 cuboid. The output of the kth filter in the first convolution layer is given by

$${\hat{R}}^{\left[1,k\right]}\left(0,w,0\right)=\;\sum_{z=0}^{S}\sum_{v=-b}^{b}f^{\left[1,k\right]}\left(0,u,z\right)R\left(0,w+v,z\right),\;w=0,1,\ldots ,\left(W-1\right).$$

A bias is added to each filtered output and passed through the ReLu activation function. The $K_{1}$ filtered outputs are combined into a $1\times W\times K_{1}$ cuboid. The width of the cuboid is adjusted if a pooling layer follows the convolution layer. Subsequent convolutions are also unit height cuboid convolutions which result in vectors which are then combined into unit height cuboids. An FCN with softmax outputs is implemented after the last pooling layer, and a target stimulus is assigned to class $\omega_{*}$ using the rule in Equation (3).

## 4. Target and Context Stimuli

The experiments described in the next section are aimed at demonstrating various aspects of the CSD principle and the CRE applied to the recognition of ambiguous stimuli. That is, the CINET(S) should yield the expected results in various contextual environments. In the process of doing so, it is also shown that the CINET(S) model parameters can be manipulated to:

(a) Simulate various context environments;
(b) Vary the strengths of the target-context relationships; and
(c) Introduce ambiguities in the stimuli.

**Figure 4:**
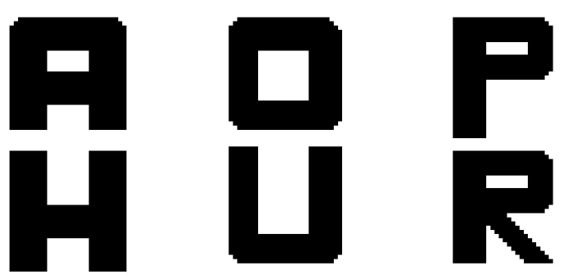
The six target letters.

**Figure 5:**
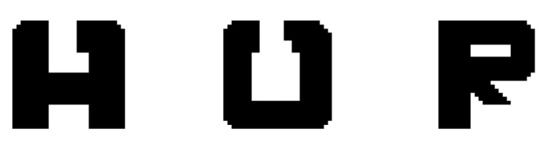
The three ambiguous letters [A/H], [O/U], and [P/R].

As noted in the introduction, the letter recognition problem was selected simply because it is suitable for demonstrating the properties of the CINET(S) classifiers by forming meaningful words. The experiments involved the recognition of six (L=6) binary letters which were digitized into 32 × 32 two-dimensional arrays. The six target letters are shown in Figure 4, and the three ambiguous letters are shown in Figure 5. The first ambiguous letter is labelled as [A/H] because it can be interpreted as target letter A or H. Similarly, [O/U] may be interpreted as target O or U, and [P/R] may be interpreted as target P or R. The specific goal, therefore, is to determine how accurately an ambiguous letter can be classified into one of its two possible interpretations with and without context. The context was incorporated by adding letters to both sides of the target letter to create a context-augmented letter set.

### 4.1. Ambiguity Manipulation

**Figure 6:**
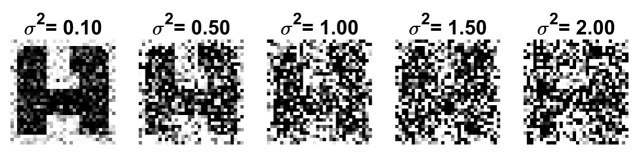
Examples of [A/H] with noise variance $\sigma^{2}$ ranging from 0.1 to 2.

There are several methods for generating test sets with varying levels of ambiguity. For example, target letters can be distorted by adding segments to the letter limbs, deleting segments, and skewing segments. Ambiguity can also be introduced by blurring or changing the resolution of the images [9,10,11]. These methods, however, are not suitable for generating large sets and are also difficult to quantify. We introduce a method to systematically increase the ambiguity level by adding increasing levels of zero-mean Gaussian noise to the noise-free pixels of the letter arrays. The noise $N_{j}$ added to $T_{j}$ is specified by the variance $\sigma_{j}^{2}$. Because the noise is random, a large set of distorted characters can be generated for a given $\sigma_{j}^{2}$. Ambiguity is increased by increasing $\sigma_{j}^{2}$. Examples of noisy images of the ambiguous letter [A/H] with noise levels in the range used in the experiments are shown in Figure 6. Observe that the letter [A/H] is difficult to recognize visually when the variance is greater than 1.5. The effect of distortions and noise on the other ambiguous letters is quite similar.

### 4.2. Context Manipulation

The context environment during testing can be congruent (same) or incongruent (different) from the context environment that was used during training. Incongruencies can be generated in many ways. The most obvious is to replace the context stimuli that were used during training with different stimuli during testing. However, this method is not suitable for resolving ambiguous stimuli because the correct interpretation of ambiguous stimuli in the new environment is unknown. Our definition of incongruent, therefore, includes weakened and/or impaired context stimuli, but does not include replacing the context with different stimuli. Other possibilities to manipulate context include changing the positions and orientations of the context letters [9], and the colors of the context backgrounds [17]. We introduce a method to generate large test sets with quantifiable incongruencies by manipulating the CINET(S) model parameters $\alpha_{j+m},\;m\neq 0$ and $N_{j+m},\;m\neq 0$ individually, or together. The context weights can be decreased to emulate weak target-context relationships. The noise in the context stimuli can be increased to emulate impaired context. The context environment can also be manipulated by varying the weights and noise simultaneously.

**Figure 7:**
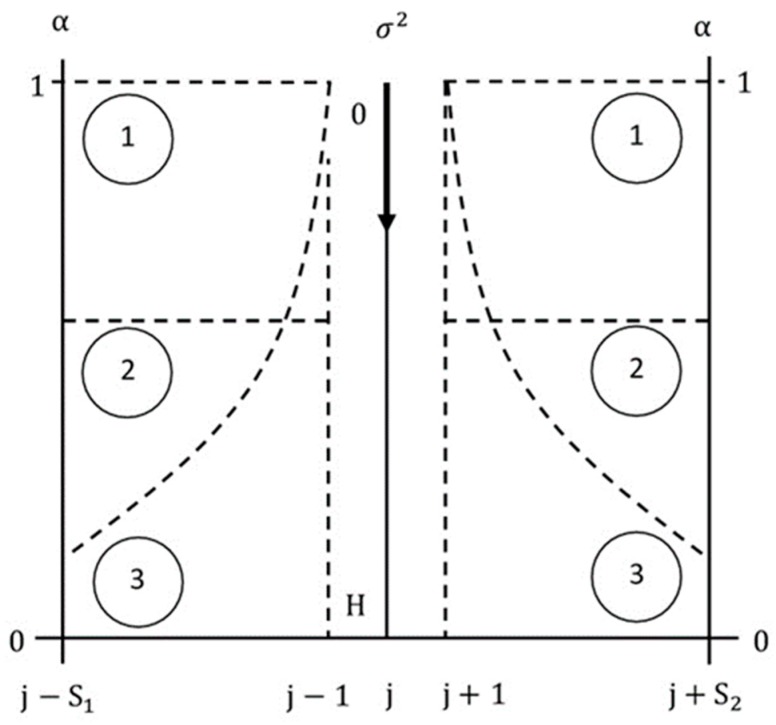
Ambiguity and context manipulations. The context strength is decreased by decreasing α, context noise is increased by increasing $\sigma^{2}$, $S_{1}$ and $S_{2}$ are the number of left and right context stimuli, respectively, and j is the spatial or time index.

Figure 7 summarizes ambiguity and context manipulations through the introduction of random noise and setting of context weights, respectively. The figure shows the left and right context strength axes (α), as well as the ambiguity level axis $\sigma^{2}$ in the center. The range of α is from zero to one, and the range of $\sigma^{2}$ is from zero to H (high), where H is a suitably high value. Three symmetrical target-context strength patterns are displayed. The horizontal line labelled 1 corresponds to full target-context strength across the entire span, the horizontal line labelled 2 corresponds to a weaker context strength across the entire span, and the variable curves labelled 3 show a decaying influence of the context as the span increases. The arrow pointing downwards on the ambiguity axis indicates the ambiguity level. The length of the arrow is proportional to the ambiguity level introduced by adding random noise to the letter.

### 4.3. CNN Architecture

**Figure 8:**
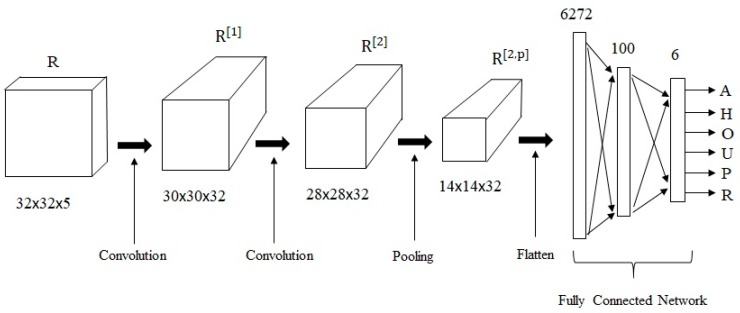
The architecture of the CINET(4) model used in the experiments.

The CNN architecture used in the experiments consisted of a convolution layer, convolution layer, pooling layer, and a 2-layer FCN, in which the first layer used sigmoidal activation functions and the output layer used softmax activation functions. The “valid” operation was used in the convolution layers, and max pooling was used in the pooling layer. Figure 8 shows the architecture of the CINET(4) model implemented for [H= 32 × W = 32 × (S+1)=5] input cuboids, where H and W are the height and width of the target and context letters, respectively, and S is the context span. The number of filters were 32 and 32 in the first and second convolution layers, respectively. The filter dimensions in the first and second convolution layers were (3×3×5) and (3×3×32), respectively. The size of the pooling filter was 2 × 2. The strides of the convolution and pooling filters were set to 1 and 2, respectively. The dimension of the flattened output from the pooling layer was 14 × 14 × 32 = 6272. The number of neurons in the first and output layers of the FCN were 100 and 6, respectively. The networks were implemented using the Keras library [39,40,41].

## 5. Classification Experiments and Results

In the experiments that follow, the context-free CINET(0) and context-integrating CINET(S) classifiers were trained to classify only the target letters. The training sets were generated by adding a small level of random noise ($\sigma^{2}=0.001$) to the noise-free target and context letters. The inclusion of noise at such small levels introduces minor variations in the letters; therefore, the resulting training sets are referred to as “noise-free training sets” in the experiments. The networks were initialized with random weights and training was terminated when the cross-entropy fell below 0.001. The CINET(0) classifier was tested on the three ambiguous letters in varying noise levels. The CINET(S) classifiers were tested on the three ambiguous letters in congruent and incongruent environments. In the experiments conducted, a total of one hundred distorted and noisy versions of each ambiguous letter were generated to form the test set at each noise level $\sigma_{j}^{2}$. Because the classification results of a CNN are dependent on the initial weights, a total of thirty CNNs were initialized with random weights. The performance of each network was evaluated using the test sets. Consequently, the total number of tests conducted for each ambiguous letter at a given noise level was 30 × 100 = 300. The results for each ambiguous letter were averaged across the 300 tests, and the final classification probability was given by averaging the averaged results of the ambiguous letters. The following experiments were designed:

*Set 1: Context-free classification with the CINET(0) classifier*

The first set of experiments was aimed at demonstrating the performance of the classifier when no context is integrated into the training and testing phases. A CINET(0) classifier was trained to classify the six noise-free isolated target letters {A,H,O,U,P,R}, shown in Figure 4, and was tested with the three isolated, ambiguous letters {[A/H], [O/U], [P/R]}, shown in Figure 5. The noise level in the test ambiguous letters was varied from 0.1 to 2. It is important to note that in the absence of context, the true class of the ambiguous letter is unknown. An ambiguous letter could be classified into any one of the six classes, however, the interest is mainly on estimating the probability of an ambiguous letter being classified into one of its two possible categories. The classification probabilities are summarized in the row labeled Set 1 in Table 1. The classification probability, in this case, can be interpreted by considering the first entry in the Set 1 row of the table, which shows that the average probability of classifying the three ambiguous characters into their possible classes was 0.48 when the noise level was 0.1. That is, the average of the probabilities of classifying [A/H] as an A or an H, [O/U] as an O or a U, and [P/R] as a P or an R was 0.48 when the noise level was 0.1. Observe that the probabilities drop as the noise increases because it becomes increasingly difficult to classify each ambiguous letter into one of its two possible classes. 

*Set 2: Training and testing the CINET(2) classifier with congruent context*

**Figure 9:**
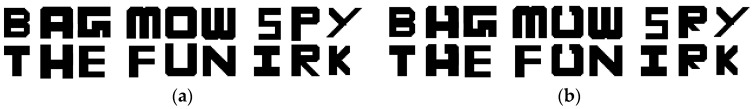
The training and test sets for the CINET(2) classifier (**a**) the noise-free context-augmented training set (**b**) the test set with noise-free congruent context.

These experiments were aimed at demonstrating the improvement in performance when strong context (unity weights) is incorporated in training, and the same noise-free context (congruent) is used during testing in order to emulate learning and testing in the same environments. A symmetrical CINET(2) classifier $\left(S_{1}=S_{2}=1\right)$ with unity weights $\left(\alpha_{j-1}=\alpha_{j+1}=1\right)$ was trained with the noise-free context-augmented training set {B**A**G, T**H**E, M**O**W, F**U**N, S**P**Y, I**R**K} to classify the six target letters in the center. That is, B and G were the context for target stimulus A, T and E were the context for target H, and so on. The training set is shown in Figure 9a. The classifier was tested with the center letters replaced with ambiguous letters, as shown in Figure 9b. That is, the test set was {B[**A/H**]G, T[**A/H**]E, M[**O/U**]W, F[**O/U**]N, S[**P/R**]**Y**, I[**P**/**R**]K}. The same noise levels used in Set 1 were added to the center ambiguous letters. Correct classification occurred if the noisy, ambiguous letter, surrounded by congruent context letters, was resolved correctly. The results, shown in the row labelled Set 2 in the table, can be interpreted by considering the first entry, 0.99, which shows that when the noise level was 0.1, the average of the probabilities of correctly classifying [A/H] as an A when the input was as B[**A/H**]G, [A/H] as an H when the input was T[**A/H**]E, [O/U] as an O when the input was M[**O/U**]W, [O/U] as an U when the input was F[**O/U**]N, [P/R] as a P when the input was S[**P/R**]Y, and [P/R] as a R when the input was I[**P**/**R**]K, was 0.99. By including context, the CINET(2) classifier has the ability to resolve stimulus ambiguities quite effectively. As expected, the classification probabilities dropped when the noise levels increased. 

*Set 3: Training and testing the CINET(4) classifier with congruent context*

**Figure 10:**
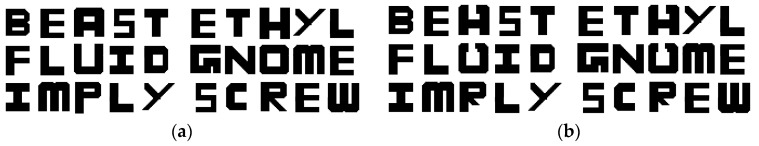
The training and test sets for the CINET(4) classifier (**a**) the noise-free context-augmented training set (**b**) the test set with the noise-free ambiguous center letters and noise-free congruent context.

These experiments were aimed at demonstrating the improvement in performance when additionally strong context (unity weights) is incorporated in training, and congruent context is used during testing. A symmetrical CINET(4) classifier $\left(S_{1}=S_{2}=2\right)$ with unity weights $\left(\alpha_{j-2}=\alpha_{j-1}=\alpha_{j+1}=\alpha_{j+2}=1\right)$ was trained with the context-augmented training set {BE**A**ST, ET**H**YL, FL**U**ID, GN**O**ME, IM**P**LY, SC**R**EW} to classify the six target letters in the center. The training set is shown in Figure 10a. As in Set 2, the classifier was tested with the center letters replaced with ambiguous letters. Figure 10b shows the test set with the noise-free ambiguous letters surrounded by noise-free congruent context. The test results under varying noise levels in the ambiguous letters are shown in the row labelled Set 3 in Table 1. The probabilities are interpreted just as they were for Set 2. It is clear that, for the same range of noise levels, the performance of the CINET(4) classifier is much better than the CINET(2) classifier. It could, therefore, be concluded that incorporating additional context improves the classification of ambiguous letters.

*Set 4: Testing the CINET(4) classifier with weighted incongruent context*

**Figure 11:**
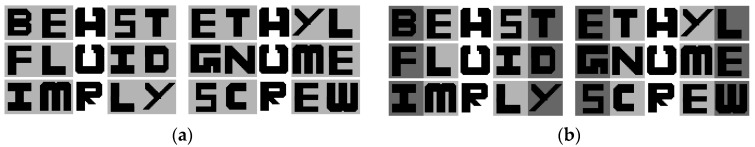
The noise-free test sets for the CINET(4) classifier with weighted incongruent context (**a**) context weights: $\alpha_{j-2}=\alpha_{j-1}=\alpha_{j+1}=\alpha_{j+2}=0.7$ (**b**) context-weights: $\alpha_{j-2}=0.4,\alpha_{j-1}=0.7,\alpha_{j+1}=0.7,\alpha_{j+2}=0.4$.

This set of experiments was aimed at demonstrating how the weights can be manipulated to simulate incongruent testing environments and to show how the performance is affected by varying the context weights during testing. The CINET(4) classifier designed in Set 3 was tested with two different sets of context weights. The first set of weights, $\left(\alpha_{j-2}=\alpha_{j-1}=\alpha_{j+1}=\alpha_{j+2}=0.7\right)$, were selected to show how the attenuation of context affects the performance. The next set of context weights, $\left(\alpha_{j-2}=0.4,\alpha_{j-1}=0.7,\alpha_{j+1}=0.7,\alpha_{j+2}=0.4\right)$, were selected to have a decaying influence as the separation span (spatial/temporal lag) between the target and context stimuli was increased. The resulting noise-free test sets with weighted incongruent context are shown in Figure 11. The results for this set of experiments are presented in the rows labelled Set 4(A) and Set 4(B) in Table 1, respectively. As expected, the performance declines as the incongruency in the testing environment is increased.

*Set 5: Testing the CINET(4) classifier with noisy incongruent context*

**Figure 12:**
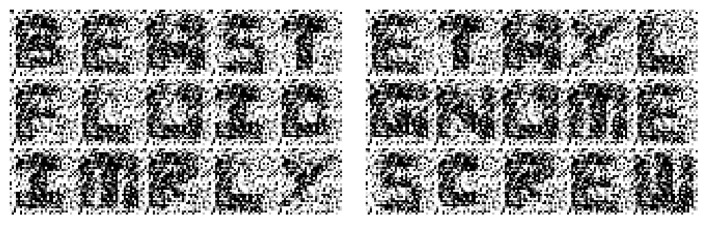
An example of a test set with noisy incongruent context for the CINET(4) classifier.

This set of experiments was aimed at demonstrating how the performance is affected when noise is added to the context stimuli during testing to generate incongruent context environments. The CINET(4) classifier designed in Set 3 with unity weights was tested with noise in the ambiguous letters, as well as statistically equivalent noise in the context letters. An example of a test set using $\sigma_{j}^{2}=0.75$ is shown in Figure 12. The results are presented in the row labelled Set 5. As expected, performance declines when context incongruency is increased by adding noise. However, the general trend observed in the Set 3 results is maintained.

*Set 6: Testing the CINET(2) classifier with flipped incongruent context*

**Figure 13:**
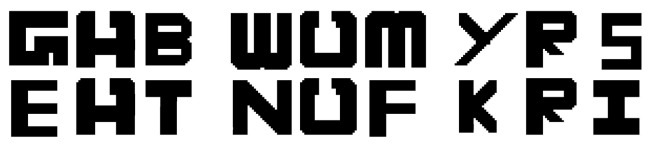
The noise-free test set with flipped context for the CINET(2) classifier.

The last set of experiments were different in the sense that the CINET(2) classifier trained in Set 2 was tested with “flipped” context to demonstrate how performance is affected if the incorrect context is used during testing. The test set, therefore, was {G[**A/H**]B, E[**A/H**]T, W[**O/U**]M, N[**O/U**]F, Y[**P/R]**S, K[**P**/**R**]I}. The test set is shown in Figure 13, and the results in varying noise levels are shown in the row labelled Set 6. Despite the fact that the same context letters were used, the results are quite poor. This, however, is not unexpected because the temporal pattern of the context was changed.

**Figure 14:**
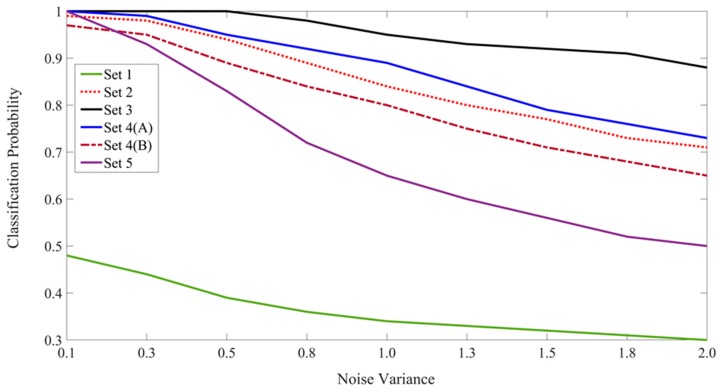
The plot of the classification probabilities for the six sets of experiments involving the classification of ambiguous letters.

The best result at each noise level is shown in boldface font in Table 1. For comparison purposes and to observe the trends, Figure 14 summarizes the correct resolution probabilities from the experiments of Sets 2–5. The Set 1 results are also included in the figure to serve as the context-free reference. The results from Set 6 are not included in the figure. 

**Figure 15:**
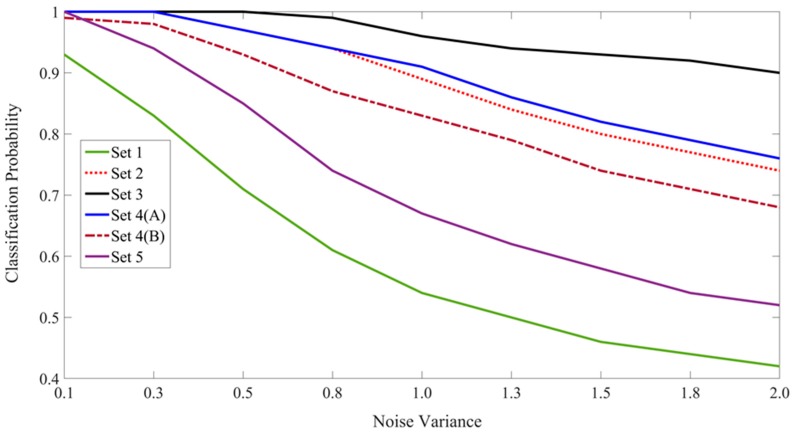
The plot of the classification probabilities for the six sets of experiments involving the classification of non-ambiguous target letters.

Although not the primary focus of this study, the CINET(S) model can also be used to design experiments to demonstrate the influence of context on the recognition of non-ambiguous target stimuli in varying congruent and incongruent environments simply by testing the targets instead of the ambiguous stimuli. In general, it can be expected that the performance will be improved by including the congruent context in the learning and recognition phases. This was confirmed by repeating all six experiments in which the target letters {A,H,O,U,P,R} were tested. The average classification probabilities are summarized in Table 2 and Figure 15. The best results are shown in boldface font. As expected, the classification probabilities are higher for non-ambiguous targets. By comparing Figure 14 and Figure 15, it is interesting to observe that the performance trends for the classification of ambiguous and non-ambiguous stimuli are quite similar.

### Conclusions from the Experiments

The results in Table 1 and Table 2 and the trends in Figure 14 and Figure 15 show that the CINET(S) classifiers perform in a desirable manner in the sense that various aspect of the CSD principle and the CRE are demonstrated. That is, congruent context helps resolve classification ambiguities, and this ability decreases as the ambiguity levels and context incongruencies are increased. The CNN offers an effective method for extracting features that are coupled across the target and context stimuli. Moreover, the random stimulus noise and context weights offer an effective way of manipulating the relationship and strength of the coupling.

The six sets of experiments and the results obtained demonstrate, quite effectively, the performance trends of the CINET(S) classifier. It can be expected that other forms of ambiguity and context manipulations will result in similar trends. Furthermore, similar results would be obtained even if the letters used for context did not form meaningful words, as long as the same context letters were used for both training and testing. Also noteworthy is that the use of simulated ambiguities and context environments enabled the systematic and quantifiable evaluation of the CINET(S) classifier model under a wide range of conditions. Clearly, such extensive experimentation and evaluation would not be possible with real data unless an enormously large data set with quantifiable ambiguities and context is collected. Undoubtedly, the CINET(S) classifier will perform similarly on real data.

Experiments can also be designed to demonstrate the influence of context on perceiving a missing stimulus, for example, a missing letter in a learned word. Because the stimulus index j can be temporal, the model can also be applied to resolve ambiguities in sequentially occurring events, such as garbled words in a sentence. Context that is not inherently sequential can also be accommodated in the model. For example, if the background of an object in an image is regarded as the context, the image can be segmented into two components: object (target) and background (context). The input to the CINET(S) classifier would then be a concatenation of the target features and context features. Finally, it is important to note that the target and context stimuli can be from mixed modalities (e.g., visual and auditory stimuli) for multisensory target-context integration, which is yet another way to combine multisensory information in brain-inspired classification systems [42].

## 6. Conclusions

The key contribution of this study is the development of a versatile, brain-inspired deep learning classifier model that can effectively resolve classification ambiguities by incorporating bidirectional weighted context during training and by using congruent context during classification. Supporting contributions include the design of a series of experiments which show that the model can emulate various aspects of the CSD principle and the CRE as applied to the recognition of ambiguous stimuli. The experiments also demonstrate the ability of the CINET(S) classifier model to introduce ambiguities due to distortion and noise, simulate various context environments, and vary the strengths of target-context stimulus relationships. Furthermore, it was noted that the model could accommodate symmetrical and asymmetrical context, is applicable to spatial and temporal context, includes the context-free classification model as a special case, and is not restricted to any particular type of classifier. The model was also used to demonstrate improvements in the classification of non-ambiguous target stimuli through the inclusion of context. The fact that the inclusion of context resolves ambiguities and improves classification is notable because context is seldom considered in the design of machine learning classification systems. Therefore, whenever possible, context should be incorporated to improve the performance of classifiers.

## Figures and Tables

**Table 1:** Classification probabilities for the six sets of experiments involving ambiguous letters.

	Noise Variance								
	0.1	0.25	0.5	0.75	1	1.25	1.5	1.75	2
Set 1	0.48	0.44	0.39	0.36	0.34	0.33	0.32	0.31	0.30
Set 2	0.99	0.98	0.94	0.89	0.84	0.80	0.77	0.73	0.71
Set 3	**1.00**	**1.00**	**1.00**	**0.98**	**0.95**	**0.93**	**0.92**	**0.91**	**0.88**
Set 4(A)	**1.00**	0.99	0.95	0.92	0.89	0.84	0.79	0.76	0.73
Set 4(B)	0.97	0.95	0.89	0.84	0.80	0.75	0.71	0.68	0.65
Set 5	1.00	0.93	0.83	0.72	0.65	0.60	0.56	0.52	0.50
Set 6	0.307	0.298	0.280	0.270	0.261	0.253	0.246	0.240	0.238

**Table 2:** Classification probabilities for the six sets of experiments involving target letters.

	Noise Variance								
	0.1	0.25	0.5	0.75	1	1.25	1.5	1.75	2
Set 1	0.93	0.83	0.71	0.61	0.54	0.50	0.46	0.44	0.42
Set 2	**1.00**	**1.00**	0.97	0.94	0.89	0.84	0.80	0.77	0.74
Set 3	**1.00**	**1.00**	**1.00**	**0.99**	**0.96**	**0.94**	**0.93**	**0.92**	**0.90**
Set 4(A)	**1.00**	**1.00**	0.97	0.94	0.91	0.86	0.82	0.79	0.76
Set 4(B)	0.99	0.98	0.93	0.87	0.83	0.79	0.74	0.71	0.68
Set 5	**1.00**	0.94	0.85	0.74	0.67	0.62	0.58	0.54	0.52
Set 6	0.331	0.319	0.301	0.292	0.289	0.286	0.277	0.270	0.265
